# Extra Supplement.—The Nursing Mirror

**Published:** 1893-11-25

**Authors:** 


"The Hospital" Nursing Mirror.

BEING THE EXTRA NURSING SUPPLEMENT OF "THE HOSPITAL" NEWSPAPER.

[Contributions for this Supplement should be addressed to the Editor, THE HOSPITAL, 428, Strand, London, W.C., and should have the word "Nursing" plainly written in left-hand top corner of the envelope.]

### News from the Nursing World.

### CHRISTMAS FESTIVITIES.

OUR friends are beginning to talk of "Christmas Entertainments," in fact at one hospital, at least, an early date is already fixed for the patients' fête. It is therefore time for us to remind our readers that due notice of festivities and descriptions intended for insertion in THE HOSPITAL must reach the office in good time. We are delighted to spare space in our columns for chronicling as many as possible of the entertainments given to patients and nurses, but naturally they are only suitable for the Christmas season, and we cannot permit them to exceed certain definite limits. It will therefore be of mutual advantage if all members of the large circle of our correspondents will aid us by sending in their contributions immediately after each event. Any approaching festivities of which preliminary announcements are desired must be officially reported to us ten days at least before they are to take place.

### THE ROYAL NATIONAL PENSION FUND.

THIS fund is progressing by leaps and bounds. The nurses are already paying into it no less a sum than £25,000 a year. The Benevolent Fund, under Lady Rothschild's guidance, is doing an immense amount of good in a quiet way. "Our Princess," as the nurses rightly name the illustrious president of this fund, is delighted and cheered by the progress made. It is a most satisfactory feature that the actuary has so arranged the tables and regulations that those nurses who join it for a short time and then draw out their savings are not permitted to do so at the expense of those who remain. In practice, owing to the special expenditure entailed by the issue of the policy, stamp duty, and other preliminary charges, the nurse who withdraws at the end of two years finds that the interest allowed upon her money is more than absorbed by the preliminary charges. It is desirable that this should be widely known and understood, as it would be well that those nurses who have not the constancy to continue to save, should recognise the fact and refrain from joining. This would obviate trouble now caused needlessly to many other people. The only disabilities imposed by the fund relate to these few inconstant ones who seem incapable of realising the safety the Pension Fund affords to all who remain policyholders. The actuaries have now completed their valuation, and the result will be communicated to the policyholders before the annual meeting, which will be held in March, at which it is hoped many nurses will be present.

### MISPLACED CONFIDENCE.

"CAN you get me a place as hospital porter or suchlike?" "I want to get into an institution as ward-maid, will you please help me?" "I am anxious to become a nurse, as all my friends feel sure that I am cut out for one." These are amongst the requests which fill the editor's letter-box, and which generally demand an immediate answer. These are much more quickly dealt with than the young lady's epistle covering two and a-half sheets of paper and containing a great many unnecessary details yet omitting the necessary ones of name and address. This is the kind of correspondent who tries the patience of the staff, whilst she probably goes about amongst her friends complaining that she never gets any answer to her inquiries, and she's sure "journals don't attend properly to one's letters." Does she usually send insufficiently signed communications in everyday life?—or is it only in editors that she places unlimited confidence, trusting to their discovering her identity instinctively?

### OUR CHRISTMAS COMPETITIONS.

WILL our readers kindly tell their friends that the garments sent in for our Christmas Competition are distributed exclusively to the adult sick poor who spend the festival in hospital wards? Each year more requests are received than can possibly be satisfied, although we have the pleasure of handing over many very delightful parcels. The needlework being expressly done for poor hospital patients, as has been set forth in each paragraph, it is quite impossible to divert it into other channels. This should sufficiently explain our inability to comply with the requests sent from all classes of institutions devoted to different, though, doubtless, to most deserving objects. We anticipate that an immense variety of garments will this year be made by our readers for the adult sick poor who have to spend their Christmas Day within hospital walls. The parcels which are the result of our annual competition receive such a hearty welcome at each institution to which they are sent, that we hope to see them increase in bulk and in number every year. The prizes will be: (1) For the best pair of socks knitted by a nurse, 5s.; (2) for the best pair knitted by any HOSPITAL reader, 5s.; (3) for the best made flannel shirt, 10s.; (4) for the best flannel or flannelette bed-jacket, 10s.; (5) for the best flannel petticoat, 10s.; (6) for the best made and simplest shaped dressing gown made and cut out by a nurse, 20s. Long seams may be done by machine. With the view of enabling an estimate to be formed of the progress made, we shall be glad to receive the names of competitors, and to hear which of the six prizes each will enter for. All parcels must reach THE HOSPITAL Office, 428 Strand, in the first week of December, and they should bear the words "Needlework Competition" in left-hand corner of address label, and also the name of the sender.

### PECULIAR PHRASEOLOGY.

IT was said, and not contradicted, at a meeting of the Guardians at Worthing, that if they had during the recent epidemic engaged extra nurses at the usual rates, it would have cost them over £100. But the guardians were apparently much too wise to do anything of the kind, they allowed the Worthing Nursing

Association to do the work, and now they are shocked and surprised by a suggestion that they should give it a grant of £20. In preference to engaging and maintaining a district nurse of their own, the Guardians at ordinary times pay £40 per annum to the association for supplying a nurse when required, and an additional allowance of £20 does not seem unreasonable, considering the amount of work necessitated by the late awful visitation of typhoid. One Guardian, however, promptly proposed to reduce the sum by half, which was ultimately done. Another advanced the rather peculiar idea, "if, when work was done, persons applied for gratuities, it was time that, as a Board of Guardians, they should put their foot down." Now, gratuity is certainly a curiously misapplied term in relation to good work done by a society which deserves thanks, as well as just remuneration, for having relieved the Board of expense, of trouble, and of immediate responsibility.

### DRUGS VERSUS COMMON SENSE.

Inventors and vendors of soothing syrups may congratulate themselves on having far out-distanced Herod! Whilst he only commanded one wholesale slaughter of helpless babes, they still manage to swell the infant death-roll month after month and year after year. The latest reported victim died because the dose given by the mother came from a bottle which had been in the house for some time, and the poison in it had increased in potency. This is politely recorded as a misadventure; but presumably soothing syrups are often "kept standing" in private households, for even the ignorant women who find drugs "quiet" their ill-cared-for, improperly nourished infants, don't usually administer the whole bottle at once! Indiscriminate poisoning can only be stopped by every trained nurse and intelligent mother waging constant war against the iniquitous system of substituting patent soothing syrups for common-sense attention to the comforts of much-enduring babies.—

### CONFLICTING INTERESTS.

It is surely quite time that a stand should be made against the practice of requisitioning for well-to-do parishioners the services of a nurse established ostensibly to attend the sick poor. In country districts the residents do not burden themselves with the maintenance of a trained nurse until they are convinced that she is urgently needed, and therefore it is obvious that when she is permitted to go to a case at the squire's, she must neglect her work amongst the cottagers. However clever and experienced, it is beyond her powers to be "in two places at once," and therefore the round of visits is shortened, or even omitted, that the prosperous patient (a subscriber, perhaps, whom it won't do to offend) may get due attention. If the doctor has not courage in the first instance to forbid this perversion of charity, he is unable afterwards to risk offending a valuable patron by insisting on the substitution of another nurse for the district one, whom the rich patient probably declines to relinquish willingly. Even when a trained nurse is in charge of a cottage hospital, she is sometimes faced by the serious problem of either taking a private "emergency" case, to the neglect of her own special duties, or else to offend the doctor, to whom she is under obligations for pass kindnesses, and on whose favour so much of her future comfort naturally depends.

### FREE OF DEBT.

The new Nurses' Home at Blackburn Infirmary is clear of all debt, thanks to the warm and generous support it has met with from some fifty ladies and gentlemen and from money collected by means of several military and bicycle tournaments, benefit performances, &c. Such a prosperous commencement augurs well for the future of the nursing staff at this useful infirmary.

### INADEQUATE REFORMS.

"That an able-bodied inmate be placed in charge of each ward" is amongst the proposed reforms at Newton Abbot Workhouse. These two special wards are to be tenanted solely by epileptics, who certainly cannot be congratulated upon their prospects. Unless the able-bodied inmates are mentally afflicted they have no excuse for being in the workhouse, and if they be unsound in mind or body the persons nominally in their care will certainly be neglected. The so-called reforms must be thorough ones if the sick and afflicted are to be properly cared for. We presume the "two female nurses" who are to be engaged, will be fully-trained women, but that important point is not mentioned, it being merely stated that one of them is to be a certified midwife.

### THE POOR RATEPAYERS.

The sick poor in Wolverhampton Workhouse have a wise champion in Dr. Totherick, who is endeavouring to secure for them adequate attendance. At present the Board of Guardians fail to see what poor economy they are practising in overworking their one qualified nurse. Nine deaths in a week are recorded, and it wants but little imagination to see what incessant care such urgent cases of sickness must have required at her hands. The view taken of the needs of these unfortunate inmates by the doctor is happily more humane than that of a guardian, who, said "They ought to think not only of the poor in the house, but the poor ratepayers. It was the latter who were the sufferers." The last statement will not bring much consolation to paupers, who "sick unto death" may surely also claim to be described as "sufferers!"

### ANOTHER NURSE NEEDED.

A lady guardian at Leicester is calling attention to the need for another night nurse at the workhouse. At present one has to look after both sides of the building, and, remarked a gentleman, "No complaint had been received as to the night nursing." This sounds very much as if reforms might wait until "something" was definitely complained of. Bearing in mind recent inquests when the coroner has called attention to the need of qualified nurses, we should have thought that few guardians would be anxious to wait for any more fatalities to bring this necessity urgently before their own notice as well as to that of the public at large.

### SHORT ITEMS.

The Rushden Nursing Association has had its third annual meeting, at which a good record of work was placed before the subscribers.—Miss Emily Taylor, member of the Ladies' Sanitary Association, is giving some lectures on "How to Keep Well," which are thoroughly appreciated by attentive audiences in the villages in the Colne Valley district.—A scheme for regular training and instruction of probationers at the St. Olave's Infirmary has been agreed to by the Board of Guardians.—Miss Steele has been appointed nurse at the Lancaster Workhouse.—Miss Bromley, lecturer of the Ladies' Sanitary Association, has been giving addresses on "Hygiene and Nursing" in Shropshire, which have attracted much attention and collected large audiences.—Miss Lucy Scott, another of the Ladies' Sanitary Association lecturers, has been giving some successful courses on the same subjects in other parts of Shropshire.—*Nursing Notes* has entered on its seventh year of prosperous existence. The December number contains a capital article on the R. N. P. F. for Nurses.

### On the Nursing of Diseases of the Stomach.

III.—VOMITING (*continued.*)

From consideration of the mechanism and causes of vomiting it is clear that to treat a case of vomiting, it is necessary first to find out its cause. If due to the presence of irritant substances in the stomach, as indigestible food or poison, it is best to empty the stomach of its contents. A drink of warm water, or warm water with a teaspoonful of mustard in it, is a simple means of doing this. In cases of irritability of the stomach, it is advisable to give the stomach rest for a time as far as possible. This may be done by nutrient enemata, or by giving only very small quantities of milk at a time, by the mouth. It may be necessary to give small quantities that have been already digested, such as milk already diluted by adding liquor pancreaticus to it. A pint of milk and a quarter of a pint of water are warmed to a temperature of 140° F., or one-half of the diluted milk may be heated to boiling point and the other half cold, may be mixed with it. Into this two teaspoonfuls of liquor pancreaticus are added and ten grains of bicarbonate of soda. This mixture is kept warm for an hour or an hour and a half, and then boiled for two or three minutes. In cases of excessive vomiting, only a teaspoonful of milk should be given at a time.

A blister or a mustard-leaf to the epigastrium is also sometimes ordered in these cases. Sucking ice may afford relief. Sedatives are often given with advantage; effervescing mixtures, bismuth or morphia, and opium may be prescribed if not contra-indicated. Two, three, or four drops of tincture of iodine in a teaspoonful of water has been occasionally found efficacious.

In obstructive vomiting, the treatment is of course, if possible, to remove the obstruction by surgical means. In mild cases of vomiting during pregnancy, a little food should be given before the patient gets up. Constipation must not be overlooked, and gastric sedatives may probably be ordered. In severe cases the patient must be kept lying down, and small quantities of liquid food only be given at short intervals. If milk is not retained, even when iced or with lime water, Brand's Essence or Valentine's Meat Juice may be tried. If emaciation be observed, recourse will probably be had to nutrient enemata. But in all such severe cases medical aid is absolutely essential. In cerebral disease remedies which act on the stomach will probably be prescribed to relieve vomiting; also sedatives, such as bromide of potassium. Often sea-sickness defies treatment, and there is no unfailing remedy. Lying quite still with the eyes closed, so as to avoid the impressions of moving objects, may prevent the onset of vomiting. Fresh air is also important, and various drugs are, of course, recommended.

Vomiting after administration of an anæsthetic may be troublesome; it may be relieved by sucking ice, or by small quantities of strong coffee if permitted.

Pain in the Stomach.

Pain is a prominent symptom in cases of gastric disorder. It is one of the symptoms of dyspepsia, and follows as a rule the taking of food, either immediately or some hours after. But there is another kind of pain which has a relation to food, and that is a pain which comes on when the stomach is empty. This form is regarded by some as a kind of neuralgia, and it is relieved by taking food. It if comes on before the ordinary time for a meal, eating a biscuit will generally give relief, for this pain may occur when the interval between meals is only of the usual length, or if such interval is prolonged. It sometimes happens that persons who have nothing after their late dinner, wake in the night with a pain in the stomach, or uneasiness, or complain of a feeling of sickness in the morning. This discomfort is removed by the practice of taking a biscuit or some slight refreshment before going to bed, and before getting up in the morning. In speaking of vomiting, we referred to one form produced by irritant substances in the stomach, such as undigested food or poisons. These give rise to inflammation of the mucous membrane, which result in pain. There are also other causes of inflammation besides the presence of irritant substances in the stomach, also accompanied by pain. Pain is a prominent symptom in gastric ulcer and cancer of the stomach.

But pain may be referred by the patient to the stomach, and yet not be due to disease of that organ. Thus the muscles over the stomach may be the true seat of it. Just as there can be pain in the muscles in other parts of the body, so the abdominal muscles suffer. The pain is similar to that produced by much exertion or over fatigue, as the aching in the legs after much walking. Muscular pain may arise from slight causes in those who are in a weak state. Again, pain referred to the stomach may be due to disease beginning in the spine, about the middle of the back. Nerves coming off from the spinal cord extend round the body to the mid line in front. Disease in the vertebræ may cause irritation of the nerves as they are coming out of the spinal canal, and the irritation gives rise to pain felt where the endings of the nerves are distributed, that is to say, over the front of the abdomen. If children complain of pain over the abdomen it is commonly thought to be due to stomach disorder, but in all cases of persistent pain over the stomach the back should be examined. It may then be found that pressure over the spine produces pain, or that there is a projection at one part.

Another cause of persistent pains over the stomach area is disease of the large artery, the aorta, which lies at the back of the abdomen in front of the vertebral column. The walls of the aorta may become diseased, and in consequence weakened in parts. Then the blood inside pushes out the wall, making a pouch, or aneurism, and this having once been formed, the constant pressure of the blood inside the aorta tends to increase the size of the pouch. In this way a large tumour may be formed, which presses upon the neighbouring parts, causing much pain. It is much commoner in men than women, and usually occurs after middle life.

An aneurism of the aorta may rupture into the stomach, and the blood so poured out will be vomited. This vomiting of blood, or hæmatemesis, will be considered in a subsequent paper.

In nursing it is most desirable that the attendant should be constantly on the watch for fresh symptoms. By giving accurate reports to the doctor of any kind of pain of which complaint is made in his absence, she can do valuable service without unduly alarming her patient.

### Minor Appointments.

Southport Infectious Diseases Hospital.—Miss Mary Seal has been made Nurse at this hospital, she trained at Monsall Hospital, Manchester, where she also held the post of Head Nurse. We wish her success in her new work.

### Wants and Workers.

A lady requiring a comfortable home with cheerful society and at a moderate cost, can be recommended one in the house of a widow lady residing in the Midland Counties. Replies marked "Home" can be addressed to the Editor.

### Women's Work.

### FEMALE MEDICAL AID FOR THE WOMEN OF INDIA.

By Mrs. Scharlieb, M.D.

(*Read at the Women's Conference at Leeds.*)

#### II.—MEDICAL AID.

Can any service be more noble, can any career offer more reward, than that of a woman who will devote herself to the amelioration of the Hindu woman's life? Many are the women who have hearts to feel their Eastern sister's sorrows. . . . . Many who say, "Here am I; send me." But something more than warm hearts and the impulse of renunciation is necessary. . . . The noble impulse is essential, but the continuity of purpose is as essential. Our fellow-women subjects in India have many needs, and these can be supplied only by English women who have fully prepared themselves for the work.

The women are ignorant, and need education. Their surroundings are insanitary, and need to be reformed. Their bodies are liable to accident and to disease, and they need physicians. Other wants there are, and all appeal to us for relief; but to-day I speak to you only of the medical aid that is required.

The first essential is that the doctors who work among the women of India should be fully qualified. It cannot be too clearly understood that the work they will have to do is both difficult and responsible. It has sometimes been assumed that medical work among women and children is easier than it is among men. That this is a mistake must be evident when we consider that women are liable to all general diseases and accidents, while they have certain ailments and states of health peculiar to themselves. Some of these conditions are among the gravest that can exist, and severely tax the resources and courage of the most skilful surgeons. Another point to remember is that in India a woman practitioner is in a very isolated position. Her patients will not permit the presence of a male doctor—in many instances they would rather die—and therefore the medical woman has to make up her mind as to the nature of the disease, to determine on the best line of treatment, and to carry it through unaided. In other lands the young practitioner usually begins as an assistant to some senior, and even when independent practice is undertaken, consultation with someone of greater experience and skill is easily obtained. This burden of unshared responsibility and lonely work is sometimes overwhelming, and, unless a doctor is very thoroughly trained, is likely to paralyse the energies and injure the nerves.

All women intending to work in India ought to have not only the regulation training of five years, but they should if possible, hold some responsible post such as that of House Surgeon in a well-ordered hospital, before going out to India. Also all societies sending out medical women should, as far as possible, send the newly-qualified to work under a senior for a time. This arrangement is good in all ways, the junior gradually acquires experience and skill. She has time to learn something of the peculiarities of disease in India, and she learns the language of her district. The senior is relieved of much of the routine portion of her work, she learns from the newcomer many new methods and improvements, and she is relieved of the ever-present fear that a temporary illness on her part will stop and greatly injure the work for which she is responsible. She will secure her annual visit to the hills, and be able to comply with requests to visit patients in the district who are too ill to be moved into the town.

Medical women for India then should not be contented with any standard but the highest. They must remember that they need to be better trained than those who work among older colleagues, and who can easily obtain a consultant's advice.

Women who intend to be doctors in India should possess great tact and agreeable manners. The native ladies are very quick to recognize the presence or absence of these qualities, and are much more amenable to those who carefully avoid offending their prejudices and hurting their feelings. It is well for those who are to work among the natives that they should have some knowledge of the obligations of the religion, the caste, and the family life of their patients.

I have worked for years among the Hindu and Mahomedan women of Southern India, and can testify to the courtesy and friendliness with which I was always treated. Sometimes my treatment was declined, it being too violently in opposition to their customs; more often only time was needed to convince my patient and her friends that she would do well at least to try my plan. The people have a rather trying habit of expecting that any medicine or treatment will bear immediate fruit, and unless carefully warned they will discontinue the remedy which has failed to cure them in three days of a trouble that may have lasted many years.

To illustrate the necessity for tact and *savoir faire*, I may mention that the Brahmins and other high caste people have a strong prejudice against any water that has not come from a holy source—no matter how filthy—and will frequently refuse to drink medicine that has been compounded in the usual way and dispensed in bottles. In such cases the dry ingredients may often be given in the form of powders to be mixed by the patient with water or milk at the time of administration. Again, it is sometimes a matter of extreme importance to feed a patient in a manner they strongly disapprove. Thus, perhaps, chicken broth or brandy may be urgently indicated. If ordered in the usual course they will certainly not be taken, the refusal being point-blank; or the nourishment will be accepted—and poured away. Such remedies must be dispensed as if they were medicines, in bottles marked with graduated doses. Again, many orthodox Hindu women are sorely disconcerted by an unexpected visit, or by one after the hour of the morning ablutions. They are much perplexed, because to refuse to see the doctor is contrary to their instincts of hospitality, while the touch of an outsider involves a repetition of the lengthy process of bath and prayer before the morning meal can be prepared and eaten. Surely a lady with ordinary courtesy would carefully avoid all unnecessary worry for her patient.

(*To be continued.*)

### Notes and Queries.

SPECIAL NOTICE.

The contents of the Editor's Letter-box have now reached such unwieldy proportions that it has become necessary to establish a hard and fast rule regarding Answers to Correspondents. In future, all questions requiring replies will continue to be answered in this column without any fee. If an answer is required by letter, a fee of half-a-crown must be enclosed with the note containing the enquiry. We are always pleased to help our numerous correspondents to the fullest extent, and we can trust them to sympathise in the overwhelming amount of writing which makes the new rules a necessity. Every communication must be accompanied by the writer's name and address, otherwise it will receive no attention.

### Queries.

(226) *Home.*—Is there any convalescent home at the seaside where a youth of sixteen, very delicate, with tendency to scrofula, could be sent? His widowed mother could pay five shillings a week for some months.—*Mrs. McCraig.*

(227) *Secretary.*—Ought I to regard with suspicion a proviso that I should invest a sum of money, amounting to hundreds of pounds, in a society on accepting office as secretary of the same?

### Answers.

(226) *Home* (Mrs. McCraig).—The Sea-bathing Infirmary at Margate would be admirable for the case. Write to the Secretary and ask terms, and then try if you cannot get friends to supplement the mother's payments. Other homes are given in Burdett's Hospital Annual, 428, Strand, London, W.C.

(227) *Secretary.*—Most certainly. The police courts have of late years proved this to demonstration.

### Nursing in the United States.

(By Miss Darche, Lady Superintendent New York Training School for Nurses, Blackwall Island.)

### PROPER ORGANISATION OF TRAINING SCHOOLS.

(*Concluded from page* lxv.)

One of the first points to determine in training-school organisation is the relationship the school management shall maintain to the hospital management, whether the school will be under a separate board or committee, or whether it will be managed by the hospital board or committee. In the first case, a contract will be entered into, which will make the school management responsible to the hospital committee for the proper nursing of the hospital; in the second case, the head or superintendent appointed by the hospital board or committee to be over the nursing department will be held responsible for the proper nursing of the hospital. Good schools are conducted upon either plan, the point being to have it distinctly understood that the nursing is considered a department in itself, and is to be organised and managed as such.

This vital point having been settled, and a superintendent appointed, several questions at once arise as to her jurisdiction. What shall her relationship towards the heads of the other departments of the hospital be? What the limit of her prerogatives? What her authority? The importance of these questions demand the highest authority, and I cannot do better than quote Miss Nightingale on these points. In her paper on "Relation of Hospital Management to Efficient Nursing," she says:—

"Vest the charge of financial matters and general supervision, and the whole administration of the infirmary in the board or committee, *i.e.*, in the officer who is responsible to that board or committee. Vest the whole responsibility for nursing, internal management, for discipline and training of nurses in the one female head of the nursing staff, whatever she may be called. The necessity of this is not matter of opinion, but of fact and experience. The training or nursing matron should be responsible to the governing authorities of the infirmary or hospital, or to any committee appointed by them for the purpose. There should be but one superintendent or training matron in the nursing department, and it follows as a matter of course that she should also be matron of the hospital. She may have a housekeeper subordinate to her, and, if the training school be large, a deputy mistress (assistant superintendent) of probationers (pupil nurses). She should be made responsible, too, for her results, and not for her methods. Of course, if she does not exercise the authority entrusted to her with judgment and discretion, it is then the legitimate province of the governing body to interfere and remove her. The matron or nursing superintendent must be held responsible for the conduct, discipline, and duties of her nurses; for the discipline of her sick wards, for the care and cleanliness of the sick, for the administration of diets and medicine, for the care of linen and bedding, and probably for the patients' clothing. The duties which each grade has to perform should be laid down by regulation, and all that the medical department or the governing body of the hospital has a right to require is that the regulation duties shall be faithfully performed. Any remissness or neglect of duty as a breach of discipline can only be dealt with to any good purpose by report to the matron (superintendent of nurses) of the infirmary. No good ever comes of the constituted authorities placing themselves in the office which they have sanctioned her occupying. No good ever comes of anyone interfering between the head of the nursing department and her nurses. It is fatal to discipline. It is necessary to dwell strongly on this point, because there has been not unfrequently a disposition shown to make the nursing establishment responsible on the side of discipline to the medical officer or to the governor of the hospital. Any attempt to introduce such a system would be merely to try anew and fail anew in an attempt which has frequently been made. In disciplining matters, a woman can only understand a woman. Simplicity of rules, placing the nurses in all matters regarding treatment of the sick absolutely under orders of medical men, and in all disciplining matters absolutely under the female superintendent, to whom the medical officer shall report all cases of neglect, is very important. At the outset there must be a clear and recorded definition of these classes of jurisdiction."

From the above it is evident that Miss Nightingale considers it is of the first importance that the question of the duties, responsibilities, and limit of authority of the head of the nursing department or school should be unmistakably understood and defined from the very beginning; and she has left no doubt in our minds as to what she considers this responsibility and authority should be.

Given a hospital, a home for nurses, and a competent head for the school, authorised to work on the lines laid down by Miss Nightingale, the development of any individual school is but a matter of time and of detail management. A definite understanding and statement should be made regarding the number of hours of daily work which will be expected of a nurse; the time which will be allowed daily for rest and study; the course of lessons and lectures which will be given; the facilities for practical training; and the outlook or prospects of future usefulness open to graduates of the school.

The subjects taught in training schools are usually anatomy and physiology, materia medica, and methods of nursing, for a first year's course; and diseases, surgery, obstetric, hygiene, sanitation, &c., for a second year's course. Usually a class receives a weekly lesson, with a course of from thirty to forty lectures, for eighteen or twenty months out of the two years; but to what extent, or in what proportion, each school determines for itself. In America many of the large schools have gradually worked up to a standard of excellence which the smaller and less important training schools look upon as the standard of training school education; but we of the larger schools are not satisfied, and know that a general standard of excellence must be arrived at before trained nursing can take that stand among the professions which it is entitled to take, but which it will never take until its training methods are more universally exact, and its course of instruction in every school more definitely and strictly laid down.

It is much to be desired that some plan might be devised by means of which a closer intercourse between training schools might be brought about. We need to co-operate in the interests of the profession; to promote what is good and to eliminate what is bad. Much has been accomplished, but much remains to be accomplished, and we must bear in mind that any successful development in the future will lie in unitedly establishing and unitedly keeping the standard of our work at its highest possible point. From its nature nursing is peculiarly a woman's work; a woman originated the training school system in England, women started it in this country, women have brought it to its present stage of development, and it is to women we must look for its future advancement.

### Appointments.

[It is requested that successful candidates will send a copy of their applications and testimonials, with date of election, to The Editor, The Lodge, Porchester Square, W.]

Glasgow Cancer Hospital (Free).—Miss Elizabeth Killen, trained at the London Cancer Hospital, Brompton, has been appointed Matron of the Glasgow Cancer Hospital, to which she takes with her the good wishes of many friends.

Southport Infectious Diseases Hospital.—Miss Kate L. Laing has been appointed Matron of this hospital. She was trained at Sir P. Dunn's Hospital, Dublin, and was afterwards Sister-in-Charge at the Hardwicke Hospital in the same city. We congratulate Miss Laing on her appointment.

### Everybody's Opinion.

[Correspondence on all subjects is invited, but we cannot in any way be responsible for the opinions expressed by our correspondents. No communications can be entertained if the name and address of the correspondent is not given, or unless one side of the paper only be written on.]

### ASYLUM v. HOSPITAL TRAINING.

Dr. William Harding, M.R.C.P.Lond., writes from Northampton County Asylum, Berrywood: While fully agreeing with the remarks in last week's Hospital that no woman can be considered an "all-round" nurse who has not hadtraining in all branches of nursing, I thing the tone of the paragraph is hardly fair to the trained mental nurse of to-day. The asylum nurse has taken up one line; her sister in the infirmary has taken up another. The latter, after three years' training (or less), is called a trained nurse and is admitted to the privileges of one, though she has never seen a mental case and is entirely ignorant of their management. The former, after full three years' training (including sick nursing of the insane), is, so to speak, still looked upon as an outsider. Is this just? I grant that an asylum nurse would be benefited by a period spent in an infirmary, but the infirmary nurse would be just as much improved by a residence for a similar time in an asylum. The two classes of nurses are occupied in different branches of their great work, but when each is competent shall we say that one is the superior? Are the qualities and knowledge required by a mental nurse inferior to those called for in an infirmary nurse? The one is as deserving of recognition and consideration as the other. She alone is the superior who can combine the experience and the knowledge of both. The mental nurse does not claim to be the same as the infirmary nurse, but is now beginning to assert that if not standing on exactly the same platform, she has her footing on one which is equally as high. It may not be generally known what is comprised in the training of an asylum nurse of to-day. I will give a sketch of the system established by Dr. Greene, the superintendent of this asylum. For the staff nurse's certificate, three years' training, including attendance upon lectures and practical instruction in the wards is necessary. Examinations, written, oral, and practical, must be passed at the end of each year, and the nurse's every-day work must be such as to show that she is a suitable woman to receive the certificate. This is given by the committee of the asylum under the signature of the chairman. The examination at the end of the first year takes in the following subjects: Elementary anatomy and physiology; first aid to the injured, and duties in emergencies; bandaging and dressing of simple wounds; making and applying of poultices, fomentations, &c. As an example I give a copy of an examination paper which is followed by an oral and practical examination.

Examination Paper for Junior Nurses.

1. What are varicose veins? What would you do in a case of bleeding from a varicose vein?
2. What changes does the blood undergo in (*a*) the pulmonary, (*b*) the general circulation?
3. What is the function of the kidneys?
4. What would you do if a patient had swallowed some poisonous liniment?
5. What is a hernia or rupture? Where are they usually found?
6. How would you distinguish between fainting and epilepsy?
7. If a patient has brought up blood, to what points should a nurse pay attention in order to assist the doctor in forming an opinion as to whether it came from the lungs or the stomach? What would you do for such a patient until the doctor arrived?
8. What would you do if a patient's dress caught fire? How would you dress a burn?

Berrywood, October 29th, 1893.

The subjects for the second examination are as follows: Ventilation, temperature, &c., of sick room; care of feeble and bedridden cases; prevention of, and attention to, bedsores, &c.; moving and carrying of helpless patients; administration of food and medicines to the sick and feeble; use of blisters, lotions, giving of enemas, &c.; taking temperatures, counting pulse and respirations, keeping clinical charts; bandaging and dressing of wounds; general knowledge of precautions to be used by the nurse in disease. I add an examination paper, which is also followed by an oral and practical examination.

Second Examination.

1. What is a bed-sore? In what class of patients are they most liable to occur? What precautions would you take to prevent them? How would you dress them?
2. What are the dangers to be guarded against in a case of rheumatic fever? To what points should a nurse pay special attention in such a case?
3. Mention some preventible causes of diarrhœa among the insane. What important points would you note in any report of a case of diarrhœa?
4. If a patient had been vomiting, to what points would you give attention in your report of the occurrence? Mention any causes of vomiting you know.
5. Give a description, as fully as you can, of the structure of the lungs. State what parts are affected in the following diseases: (*a*) Pneumonia, (*b*) pleurisy.
6. State what precautions should be taken in nursing a case of scarlet fever. Why are these precautions necessary? How is the disease generally spread?

Berrywood, November 14th, 1893.

The third examination includes the nursing of the acutely insane and the general duties of the asylum nurse. The examination is conducted by the Medical Superintendent, and, jealous of the reputation of his asylum, he is not likely to submit to his Committee the name of any woman whom he does not deem worthy of the certificate. I would ask whether it is just to say that the woman who has passed through this training satisfactorily is not as much entitled to be called a trained nurse as her sisters from the infirmaries.

### "A SEARCHING INVESTIGATION IMPERATIVE."

"Fairplay" writes: In the interests of justice I wish to make some statements with regard to the case recently tried at the Manchester Assizes, viz., Fincken v. The Royal Infirmary. I have made myself acquainted with all the details of the case, and consider that the counsel employed by the defendants should have stated the cause of plaintiff's dismissal. The latter left her patients, amongst whom there was a woman dying of small-pox, without the consent of the Lady Superintendent of the Royal Infirmary, a proceeding calculated to bring discredit to the latter institution. What medical man or private individual would care to employ a nurse who had acted in such a manner? I consider that a woman who would leave a dying patient in the hands of inexperienced people has mistaken her vocation; she is not fit to be a nurse, and her behaviour has been against the common laws of humanity. I have resided for some years in Manchester and have always heard that the officers of the Royal Infirmary are careful as to where they send their private nurses, in order that the latter may not be placed in an uncomfortable position, but surely, in the case in point, it was the duty of the nurse to remain at her post until the Medical Officer of Health succeeded in providing a substitute. It was stated that Nurse Fincken left the case because she was ill. If it were so, no medical man would have asked her to remain. Moreover, when she accompanied the other nurse to Monsall Hospital she did not report herself as ill to the Resident Medical Officer, but introduced her companion as "the sick nurse." The Lady Superintendent, under the circumstances, could not do otherwise than dismiss her. Legally the nurse was entitled to her certificate in consideration of her three years' service, but as she was dismissed before her examination could take place, it could not be expected that the authorities would allow her to return to the Infirmary for that purpose. It is to be hoped that the Board of the Infirmary will in the future insert some clause in their rules whereby the certificate can be refused to a nurse (even if her three years' training has been completed) who should be guilty of a grave breach of discipline. I should like to know if it is the opinion of the readers of The Hospital that sick people are to be left without a nurse because they are poor and their surroundings uncleanly?

### Our Examinations.

"Use a little disinfectant" is certainly too vague phraseology for a nurse ever to permit herself. Yet more than one of our correspondents has indulged in sentences very little more definite this month.

Accuracy is of the greatest value in such important matters as the one now before us, and it is always satisfactory to have the strength and description of the disinfectants recommended distinctly specified. So many deodorants are accepted by the public as disinfectants that no qualified nurse should ever lose an opportunity of stating definitely what she has been taught to consider the best to use, subject always to the sanction of the doctor in attendance. A great many of our competitors have given very good hints of what should be done for cholera cases until the doctor comes. They all seem sufficiently impressed with the necessity for isolation. However it sounds a little too autocratic to write that all unnecessary articles, as well as the patient's friends, should be immediately removed from the room! Such high-handed measures ought to be pursued with reservations. If a nurse did not act with rather more tact and discretion she might find herself very unwelcome in the dwellings of the poor.

In directions as to the removal of excreta most writers dwell on the disinfection of the pan, &c., but hardly one gives adequate warning respecting the danger of using ordinary w.c.'s and the precautions needful. We regret this omission, because a nurse's responsibilities include the safe bestowal of everything which leaves the room of a cholera patient, and she should understand and practise extreme precautions in every detail. In the proper disinfection of bedding no needless destruction of this, any more than of clothing, should take place.

### Examination Question for November.

If a case of cholera occurs in a cottage or in workmen's dwellings, what precautions should be taken by the nurse summoned to the case, (*a*) as regards the patient, (*b*) the rest of the household, and (*c*) herself?

### Prize Answer.

*a.*—The doctor should be sent for without delay, and in the meantime the patient must be put to bed between blankets, with a hot bottle to the feet. He should be kept quite warm, and if troubled with pain or cramps hot flannels should be applied to the abdomen. If thirst be excessive, sips of cold or iced soda water, or soda and milk, may be given. The patient should be kept perfectly quiet and soothed until the arrival of the doctor, whose instructions must then be carried out implicitly.

*b.*—As cholera generally sets in suddenly, and all the evacuations are infectious in the very highest degree, the rest of the household must be protected as much and as quickly as possible from infection: (1) By not allowing any member of the house near the patient; (2) by receiving all evacuations into vessels containing about a wineglassful of a saturated solution of sulphate of iron—copperas—(a nurse should take this with her if she knows she is going to a case of even suspected cholera); and (3) all soiled clothing should be thoroughly disinfected and boiled, or, safer still, burnt, and all mattresses used should be burnt.

*c.*—The nurse must wear washing dresses, petticoats, and stockings. She should wash her face and hands and scrub her nails in carbolised water (1-20) before taking food, and she should never eat in the sick room.

M. L. Ashton Warner.

### Examination Question.

Describe the nursing of a case of influenza, and state what a nurse should specially watch for.

Answers must be sent in by December 5th, written only on one side of the paper, accompanied by full name and address, directed "Nursing," care of Editor of The Hospital.

### Causerie from New England.

### HONOUR FOR THE BOSTON CITY HOSPITAL.

The exhibit sent by the Boston City Hospital to the Columbian Exposition, at Chicago, has received a medal, the highest honour bestowed by the Committee of Awards. This exhibit included a large collection of plans and photographs representing the varied work of the hospital in surgical, medical, infectious, and out-patients' departments. Large bound volumes contained all the printed matter, reports, rules, notices, &c., relating to the hospital *regime*, including the important work of its training school for nurses. A considerable number of models showed furnishings, utensils, &c. which were either invented or adapted by the hospital staff or officers. The exhibit was the outgrowth of much time and thought, and made a felicitous object lesson to demonstrate intelligently the practical working of the institution. Before going to Chicago, it was set up in the medical library of the hospital, where it attracted a large number of visitors. The entire exhibit is now on its way back to Boston, where it will form the basis of the medical museum soon to be established at the hospital. The intention of this museum is to be one of the class rooms for the instruction of the training school pupils in practical work, in the same way as a laboratory serves to educate. It also has a helpful value in preparing graduates to become matrons and superintendents of nurses in other hospitals. The management expects to increase the museum on lines already indicated, and to add new features from time to time.

### Where to Go.

Nurses Residential Club will be opened by the Duchess of Portland on December 6th, at 17, Nottingham Place.

Bloomsbury, 29, Queen Square. Art for Schools Association gives its annual exhibition of framed and unframed pictures, November 28th to December 11th, admission free.

Trained Nurses Club, 12, Buckingham Street, Strand—a sale of work in aid of the funds of the institute will be held on 29th and 30th November, commencing at two p.m.

Westminster Town Hall.—Admsssion free, People's Concert Society, high class music, every Sunday evening at seven o'clock.

St. Martin's Town Hall.—The Children's Salon sale of work will take place on 20th and 21st December. The aritstic work which has been made by children will be sold for the benefit of the cot at the North West London Hospital.

Holy Redeemer Mission House, Wilmington Square, Clerkenwell. The Sisters of Bethany, who work in this very poor district, are appealing earnestly for funds to help them to relieve the urgent wants of their neighbours.

The Dudley Gallery. — The New English Art Club has once more opened its doors at the Dudley Gallery, Piccadilly. This year's level is a very high one, and much admiration is heard on all sides. The selecting jury have been commendably difficult to satisfy, and the comparatively few works they have admitted from the hundreds submitted to them all justify their retention. The New English Art Club is the only corporation of artists which administers the same law to itself as to outsiders. The fact of membership does not carry with it the right to exhibit at its bi-annual shows. Members as well as non-members must submit their intended exhibits to the selecting jury. It is obviously impossible to dwell in detail on the merits of each picture. The most prominent successes are Mr. Francis Bates' "Limekiln," Mr. Wilson Steer's magnificent study of a beautiful girl in an unconventional dress, and Mr. George Thomson's successful rendering of the poetic hour "'Tween the Lights"; when, though the lamp burns bright within doors, the undrawn curtain shows an enchanting glimpse of twilit river without. Mr. Walter Eickert occupies the place of honour, and never proved more conclusively that he is a veritable master. But now he is weaned he shows a remarkably vigorous and original style of his own. His picture is a marvel of technique, with none of the dulness which scientific experiments usually produce on the mind of the uninitiated, for his out-door groups in their delicious costumes have all the airy grace and charm of Watteau.

### The Muses' Looking Glass.

### "UTOPIA" AT THE SAVOY THEATRE.

"UTOPIA (Limited), or the Flowers of Progress," written by W. S. Gilbert, and composed by Sir Arthur Sullivan, is a huge success, and no wonder. The plot is most bright and original, the dialogue up-to-date, and the songs charming, either by their humour or their melodiousness. As the curtain rises a procession of Utopian maidens enters. Their charming robes are of shaded silks of rainbow tints, and their deep red locks reach nearly to their feet and are twined with flowers. They sing the joys of Lazyland. The King's ministers enter and discuss the Constitution. Says Tarara (the Public Exploder), "By our Constitution we are governed by a despot, who, although in theory absolute, is, in practice, nothing of the kind—being watched day and night by two Wise Men, whose duty it is, on his very first lapse from political or social propriety, to denounce him to me, and it then becomes my duty to blow up his Majesty with dynamite—and, as some compensation to my wounded feelings, I reign in his stead."

The King is expecting the return of his daughter Zara, who has been five years in England, and who now returns with a picked retinue, who are each to introduce such improvement as will convert Utopia into an England, only more so. Thus Captain Fitzbattleaxe undertakes the army; Sir Bailey Barre—

An eminent Logician who can make it clear to you
That black is white—when looked at from the proper point of view—

reforms the laws, Lord Dramaleigh is to be Lord Chamberlain, and Mr. Goldbury, a company promoter, is to set the kingdom on a sound financial basis, to achieve which he suggests that the island should be run as a joint stock company, *limited*, for,

If you come to grief and creditors are craving
(For nothing that is planned by mortal head
Is certain in this vale of sorrow—saving
That one's Liability is Limited)—
Do you suppose that signifies perdition?
If so you're but a monetary dunce—
You merely file a Winding up Petition,
And start another company at once!

The second act opens with the new *régime*, the nymphs have discarded their picturesque disorder and appear in most truly exquisite "drawing-room" attire, veils, bouquets and tiaras (Parisian Diamond Co's.) complete. It is a charming scene. The new Lord Chamberlain conducts the presentations in the most orthodox manner, and to those who have, as well as those who have never seen a real "Drawing-room," the scene is delightful for its beauty and the exactitude with which it imitates the real thing. True to the Utopian desire to improve on English customs, the ceremony takes place at night, and a plate of mixed biscuits is handed round to refresh the weary after their struggles to reach the Presence. It now appears that Princess Zara has lost her heart to handsome Fitzbattleaxe (whose able representative, Mr. Kenningham, is a novice to the stage; he has, hitherto, sung in a choir at Canterbury; but, strange to say, his acting is as good as his singing), to the chagrin of the two Wise Men, who each wish to marry her. Her independent behaviour is a great shock to her young sisters, who have been carefully trained by their governess, Lady Sophy, according to the tenets of fifty years ago.

How fair! How modest! How discreet!
How bashfully demure!
See how they blush as they've been taught!
At this publicity unsought!
How English and how pure!

They enter the house because "Lady Sophy has sent us in here because Zara and Fitzbattleaxe are going on in the garden in a way which no well-conducted young ladies should witness."

Lord Dramaleigh: "We are much obliged to her Ladyship."

Kalyba: "Are you? I wonder why?"

Nekaya: "Don't tell us if it is rude."

Lord D.: "Rude? Not at all. We are obliged because she has afforded us the pleasure of seeing you."

Nek.: "I don't think you ought to talk to us like that."

Kal.: "It's calculated to turn our heads."

Nek.: "Attractive girls can't be too particular. And please don't look at us like that; it unsettles us."

Kal.: "And we don't like it. At least we do, but it's wrong."

Goldbury: "But my dear young ladies—"

Kal.: "Oh, don't; you musn't; that sounds too affectionate."

Gold.: "And are you under the impression that English girls are so ridiculously demure? Why an English girl of the highest type is the best, the most beautiful, the bravest, and the brightest creature that Heaven has conferred on this world of ours. She is frank, open-hearted, and fearless, and never shows in so favourable a light as when she gives her own blameless impulses full play."

Her soul is sweet as the ocean air,
For prudery knows no haven there;
To find mock modesty, please apply,
To the conscious blush and the downcast eye.
Rich in the things contentment brings,
In every pure enjoyment wealthy,
Blithe as a beautiful bird she sings,
For body and mind are hale and healthy;
Her eyes they thrill with right good will,
Her heart is light as a floating feather, &c., &c.

Later he gives them such good advice as might be a motto for us all.

Goldbury: Whatever you are—be that;
Whatever you say—be true;
Straightforwardly act—
Be honest—in fact
Be nobody else but *you*.

The advice is taken, and with it the hearts of Dramaleigh and Goldbury, who pair off with the two younger Princesses; Fitzbattleaxe marries Zara, while the King elevates to the throne the redoubtable Lady Sophy. The Wise Men become the prey of the Public Exploder.

### Novelties for Nurses.

### IRISH LINENS.

(MESSRS. ROBERTSON, LEDLIE, FURGUSON, AND CO.)

INTENDING purchasers of household linen would do well to send to Messrs. Robertson, of Belfast, for patterns. Irish linens are of course proverbial, and the samples we have seen from this firm fully maintain their high standard, combined with economy. A 72-inch wide linen sheeting is offered at 2s. 9d. per yard, so that a full-sized pair of linen sheets is to be had for 9s. if the width is turned to length, or for 11s. 8d. otherwise. Linen for aprons one yard wide is really very nice at 1s., or for 1s. 4d. if 45 inches in breadth. Matrons of hospitals and other institutions should examine the bleached twill sheeting to be had for 1s. 4d. a yard, full width, or cheaper for cots. This material is really little less smooth and agreeable to the touch than the linen itself. For institutional and household use alike the damask cloths are most desirable. These can be had in no less than ten different sizes, ranging from 1s. 3d. to 4s. 3d. Excellent, too, are the brown hollands, which are both soft and durable in texture. For the nursery the Russia diaper can be most highly recommended, and the softest of towels are to be had wonderfully low in price. Lastly we must especially draw attention to the handkerchiefs which Messrs. Robertson seem to have made a speciality of their own. Really nice hemstitched handkerchiefs are sold at 4s. 3d. the dozen, quite good enough to form a welcome present at Christmas. Others more expensive, and of the finest quality are to be had too, but those that we have particularised are likely to satisfy even the most fastidious.

### The Book World for Women and Nurses.

[We invite Correspondence, Criticism, Enquiries, and Notes on Books likely to interest Women and Nurses. Address, Editor, THE HOSPITAL (Nurses' Book World), 428, Strand, W.C.]

THE BOOK-BILLS OF NARCISSUS. An account rendered by RICHARD LE GALLIENNE. (Published by Frank Murray, Derby.)

The idea which forms the scaffolding of this poetic little volume is both pretty and ingenious. Narcissus was a poetic youth who loved to read the best books. His mental development is made known to us by the bills for the books he bought at different periods of his life. "You have kept a diary for how many years? Thirty; dear me; But have you kept your wine bills? If you ever engage me to write that life, which, of course, must some day be written—I wouldn't write it myself—don't trouble about your diary. Lend me your private ledger. . . . I chanced one evening to come across those old book-bills of my friend Narcissus and was struck, well-nigh awe-struck in fact, by the wonderful manner in which there lay revealed in them the story of the years over which they ran." The book must be read to be appreciated, for its prose is as poetic as its hero, and would be shorn of its chief charm if abbreviated into the space at our command. What we would fain say to our readers, however, is this. Let our reading also be of the same significance as Narcissus's. Let us read books to enrich and strengthen our minds. We know it is unwise to weaken our digestions by constantly sucking sweets even though they be cough lozenges. Now and again if a cough troubles us one lozenge may not harm—so with sentimental stories. Now and again, when the mind is too weary to think, perhaps a guileless tale of love and "happy ever after" may rest our tired brain, if only by causing us to fall asleep by its commonality—but if we constantly read trash we weaken our critical digestion so that it will reject all stronger and more nourishing aliment. Let us feed our brains as we should our bodies, giving them a reasonable portion of good meat every day. A chapter, a page, even (if time lack) a paragraph of something which will nourish the mind within us; something that, when digested, goes to form part of the mind; helping us to clearer knowledge, sounder judgment, and wider sympathy with others—just as good food forms the tissues, strengthens the muscles, and gives us strength the better to perform our daily tasks. On the harm wrought by the trash which is not guileless, which, under the mask of an exciting plot or of fine sounding argument, revels in nasty details or finds specious reasons for calling black white, we have not here space to dwell. They work on the mind, yes, and on the soul, as chloral, chloroform, absinthe, and the liquor habit generally on the body; dulling perception of wrong and right, fostering and forcing to abnormal growth the most degrading inclinations of our poor weak human nature.

LADIES AT WORK. By Experts in the several Branches, with an Introduction by LADY JEUNE. (A. Innes and Co., London.)

Books with some such title as the present volume are a feature of the times; women are up and doing in all walks in life. Work, however, has not caused them to leave their characteristics behind them. Woman when impressed with herself does not rest until her public are also concerned about

### THE BOOK WORLD FOR WOMEN AND NURSES—*continued.*

her. Hence works on women's employments are very much to the fore at present. Some of the literary efforts in this line have been both crude and voluminous. This cannot be said of the work before us. For those outside the working field, be they men or women, some of its pages will be of the utmost interest. All the articles in the collection are not, it must be confessed, of equal merit, neither do all seem to be unanimous in the object for which they are written. Some are purely technical accounts of what has been done in the past, and apply rather more to the general result than to that share in it in which women have had a part. One or two give plain and practical advice, nothing more; and others take solely a wide and philosophic view of the whole question with which they deal. In noticing this inequality of handling the many fields of employment open to women, we have mentioned what we regard to be the drawback in a work which enfolds within its cover some real gems of merit. Lady Jeune's introduction is marked by admirable common-sense and full of that lofty ideal for women's work in the past and future which must tend to elevate the readers to whom she especially appeals. Following the introduction, comes the opening article by Miss Wordsworth, the Principal of Lady Margaret Hall, Oxford. Her subject is fitly entitled "Colleges for Women." But Miss Wordsworth has not limited the range of her conclusions to the four walls of a ladies' college. She canvasses the subject of female education generally. The whole article is full of interest from first to last. And originality and earnestness, if of thought, are cloaked in a no less markedly delightful style of diction. If to secure her words of wisdom alone, this volume should be possessed by all intelligent women. In her pages the homely are not despised; the studious are encouraged; the too impulsive after knowledge warned. All the teaching is reverent, womanly, able, and inspiring. "No system of education," she says, "is adequate which does not constantly keep in view the end which Bacon put before us long ago, "The Glory of the Creator and the Relief of Man's Estate"; and further, "Self-improvement, for self's sake is foredoomed to disappointment." On the other hand, the cultivation of talents, involving in some cases the pursuit of study, as a means to a higher end, is surely not a matter of choice—a thing to be done or left alone, as we please; but obedience to an imperative call from our Maker, which we cannot disregard, but which must affect in the long run not only our well-being, but that of others, known and unknown, from whom we cannot disconnect ourselves, "for we are members one of another." We would fain quote other of the many striking passages in which the essay abounds. Space, however, forbids us. Those who read it—and we hope there will be few who will not do so—will be cheered with the thought that the younger women who come under Miss Wordsworth's, influence, will have placed before them sound common sense and lofty ideals, which cannot but help to ennoble and strengthen many a generation that is to come hereafter. Following the educational standpoint come some pages on journalism by Miss F. Green. Some of the facts are very interesting, and all the hints to young journalists which are practicable are given. Practice, however, which includes failure and many a difficulty is the only real school here. The medical profession is tersely and practically treated by Dr. Caroline Latimer, poor music and "art" come off badly at the hands of authors, who have no doubt found the crippling shortness of space beyond them. The Misses H. M. and R. Wilson contribute an interesting chapter on hospital nursing, whilst the stage-teaching and one or two other branches of employment fill the remaining pages of a most interesting volume, which is of convenient size and moderate price.

